# What does it mean when people say that they have received expressions of concern about their drinking or advice to cut down on the AUDIT scale?

**DOI:** 10.1186/s12874-017-0435-0

**Published:** 2017-12-02

**Authors:** John A. Cunningham, Alexandra Godinho, Vladyslav Kushnir, Nicolas Bertholet

**Affiliations:** 1 Centre for Addiction and Mental Health, 33 Russell St, Toronto, Ontario M5S 2S1 Canada; 2 Departments of Psychiatry and of Psychology, University of Toronto, Toronto, Canada; 3 Dalla Lana School of Public Health, University of Toronto, Toronto, Canada; 4 Leslie Dan Faculty of Pharmacy, University of Toronto, Toronto, Canada; 5 Alcohol Treatment Center, Department of Community Medicine and Health, Lausanne University Hospital, Lausanne, Vaud Switzerland

**Keywords:** AUDIT, Severity of alcohol use, Mechanical Turk, Brief intervention

## Abstract

**Background:**

The Alcohol Use Disorders Identification Test (AUDIT) is a commonly used scale to measure severity of alcohol consumption that contains an item asking if anyone has expressed concern about your drinking or suggested you cut down. What does it mean when a participant says yes to this question?

**Methods:**

Participants who were 18 or older and who drank at least weekly were recruited to complete a survey about their drinking from the Mechanical Turk platform. Comparisons were made between at risk (*n* = 2565) and high risk drinkers (*n* = 581) who said that someone had expressed concern about their drinking regarding who had expressed concern. If the person expressing concern was a health professional, the participant was also asked what type of support was provided.

**Results:**

Expressions of concern about drinking were received more often by high risk than at risk drinkers. The most common type of person to have expressed concern was a relative, followed by a friend, or a marital partner. About one quarter of participants had received expressions of concern from a medical doctor or other health professional. All health professionals’ expressions of concern were accompanied by a suggestion to cut down and about half provided some additional support (the most common type of support was brief advice).

**Conclusions:**

Expressions of concern come from a variety of sources and the likelihood of their occurrence is partially related to amount of alcohol intake.

## Background

The Alcohol Use Disorders Identification Test (AUDIT) is a ten item screener developed by the World Health Organization to screen for hazardous or harmful alcohol use [1, 2]. The AUDIT has been extensively studied in diverse settings [3]. The AUDIT asks three items about quantity and frequency of alcohol intake (items 1, 2, and 3), three about alcohol dependence symptoms (items 4, 5, and 6), and four about harmful alcohol consumption (items 7, 8, 9 and 10). The AUDIT score range is 0-40. Items 1-8 offer five response options, and can bring 0 to 4 points to the AUDIT score. Items 9 and 10 have three response options, bringing 0, 2, or 4 points to the AUDIT score. The last of these harmful alcohol consumption items asks, “Has a relative or friend or a doctor or another health worker been concerned about your drinking or suggested you cut down?” (response options: No - 0 points; Yes, but not in the last year - 2 points; Yes, during the last year - 4 points).

What does it mean when participants indicate that someone has expressed concern about their drinking, or suggested that they cut down? Who are the participants usually referring to – a friend, relative or health worker? While understanding what it means when participants say that someone has expressed concern about their drinking may not be of great importance when the item is included as a part of the AUDIT composite score to screen for hazardous or harmful alcohol use, there are other situations where understanding who the participant is referring to may be of relevance. Notably, clinicians can use the AUDIT to provide feedback on the severity of alcohol consumption. For example, when the AUDIT is a component of a brief intervention, responses on the AUDIT can form the basis of a therapeutic conversation about the patient’s alcohol use and understanding who is expressing concern could be relevant to the content of the intervention. Another example is when the AUDIT is used as an outcome measure in an intervention trial [4] where one experimental condition consists of a brief intervention (in which a therapist expresses concern about the participants’ drinking) and the control group is some form of no intervention condition. In this case, the AUDIT score could be artificially inflated in the experimental condition, and reduce the chances of finding an impact of the intervention, because the participant may say that someone has expressed concern about their drinking on follow-up measures solely because they received the brief intervention. For example, the receipt of a brief intervention within the past year would bring 4 points on item 10, 10% of the maximum AUDIT score. As a score of 8 or more is considered positive for hazardous or harmful alcohol use, 4 points can have a major impact on the screening results.

## Methods

Participants from the USA and Canada were recruited through Mechanical Turk, an online platform where more than half a million people have signed up to complete surveys and other tasks [5–10]. The advertisement for the study asked for people interested in completing a survey about their drinking. Only people who currently drank alcohol were asked to participate. As a means of optimizing the quality of the data, the advertisement could only be viewed on the Mechanical Turk platform by those people who had completed at least 100 surveys through the platform and who had an approval rating of 95% or higher (i.e. fewer than 5% of tasks completed resulted in a complaint regarding the quality of the work completed) [11]. The advertisement also stated that the survey would take less than 15 min to complete and that the payment would be $1.50. Interested participants clicked on a link and answered two eligibility questions to identify those who were 18 years or older and who consumed alcohol at least weekly. Those eligible were then taken to an online consent form and, if consenting to take part in the survey, completed the survey. The online survey software collected potential participants’ Mechanical Turk ID number at the time of filling out the eligibility questions to prevent repeat attempts to complete the survey.

The survey assessed age, sex, country of residence, ethnic origin, education, marital status, employment status, and family income. Measurements of alcohol consumption and severity included the AUDIT. Those who responded ‘Yes, during the last year’ to the AUDIT item 10 asking whether someone had expressed concern about their drinking or suggest they cut down where further asked about who specifically had expressed concern about their drinking – work colleague, wife/husband/common law partner, relative, friend, medical doctor, or other health professional (multiple answers were possible). Those who indicated that a medical doctor or other health professional had expressed concern were asked if the health worker had advised them to cut down and if they were provided with some sort of support. Those indicating that they had been provided support were asked what type of support they were offered (brief advice, pamphlet, referred to a specialist, individual or group counselling, prescribed medication, arrange a follow-up) [12]. Even though AUDIT item 10 allows the participant to report on expression of concern but not over the past 12 months, we chose to limit this study to participants reporting expression of concern over the past year. This allows for a more homogenous reporting of treatment receipt and is potentially less prone to recall bias when compared to an expression of concern that may have happened any time in the life of the participant.

Later in the survey, participants were asked if they had used any type of treatment or help specifically for alcohol concerns in the past year. Treatment access was defined as past year use of Detox, Outpatient treatment, Inpatient/Residential/Day treatment, or Counselling by a professional (e.g., physician, psychiatrist, psychologist, social worker, etc.). Finally, the survey included four attention check questions to promote the likelihood of good quality response data and a question asking if the participant had provided honest responses (while confirming that a negative response would not impact on their payment or quality rating).

### Analyses

Participants who did not answer all the attention check questions correctly, or who indicated that they had not provided honest responses, were removed from the data set. The AUDIT-Consumption subscale (AUDIT-C; consists of the three items assessing quantity of alcohol intake) was calculated [13]. The AUDIT-C has been validated for online use [14]. The AUDIT-C was then categorized into low risk drinking, at risk drinking, and high risk drinking (respective scoring for Males: 1-3, 4-8, 9-12; Females: 1-2, 3-7, 8-12) and comparisons on the variables of interest were made between risk drinking categories. Note that we chose to use the AUDIT-C scale rather than the full AUDIT scale to categorize levels of risk in this study because we did not want to confound the risk categories with whether the person had received expressions of concern about their drinking.

## Results

Of the 4108 surveys, 87 (2.2%) participants indicated that they had not provided honest responses and 485 (11.8%) did not answer all the attention check questions correctly. This left a total of 3536 participants who were 18 years and older, and who drank weekly or more often, for the analyses. Of these, 72.5% (*n* = 2565) were at risk drinkers and 16.4% (*n* = 581) were high risk drinkers based on the AUDIT-C. A total of 11.8% of the full sample said that someone had expressed concern about their drinking in the past year, with a larger proportion of participants in the high risk drinking group than the lower risk groups (*X* ^2^ (2) = 436.2, *p* < .001; Low risk drinker: 1.3%; At risk: 7.7%; High risk drinker: 37.0%). As only a small number of participants (*n* = 5) in the low risk group said that someone had expressed concern about their drinking in the last year, this group was excluded from the remaining analyses. Table 1 displays the demographic characteristics of participants in the at risk drinker and high risk drinker groups. Compared to at risk drinkers, high risk drinkers were less likely to be married or to have some post-secondary education, and more likely to have a yearly family income of less than US$20,000. Almost all participants were from the USA (99%; not shown on Table). Further, high risk drinkers were more likely than at risk drinkers to have accessed treatment for their alcohol use in the past year.

**Table 1:** Demographic characteristics compared between Audit-C risk groups

	Audit-C		
	Risk Consumption (*n* = 2565)*	High risk consumption (*n* = 581)	*p*-value
Mean (SD) Age	33.2 (10.2)	33.7 (10.1)	.23
% Male	41.8	42.2	.89
% White	82.7	82.3	.86
% Some post-secondary education	73.7	57.3	.001
% Married/common law	48.1	40.6	.001
% Full/part-time employed	58.2	55.2	.19
% Family income < US$20,000	18.8	28.7	.001
% Past year treatment access^a^	6.5	18.8	.001

*Risk consumption: Males: AUDIT-C score 4-8; Females: score 3-7; High risk consumption: Males: AUDIT-C score 9-12; Females: score 8-12

^a^Past year treatment includes any use of Detox, Outpatient treatment, Inpatient treatment, or Counselling by a health care professional

Participants who said that someone had expressed concern about their drinking or suggested that they cut down, were asked who had expressed this concern. Table 2 lists the type of people who might have expressed concern, compared between the at risk and high risk drinker groups. Participants indicated that relatives were the most common type of person to express concern, followed by a friend, their wife/husband/common law partner, and then by a medical doctor. While both relatives and friends were more likely to express concern to participants in the high risk group compared to those in the at risk drinker group, both wife/husband/common law partner and medical doctors showed no significant difference (*p* > .05) in the proportion who expressed concern between the risk drinking groups.

**Table 2:** Proportion who expressed concern about drinking or suggested participant cut down in past year compared between Audit-C groups

	Audit-C		
	Risk consumption (*n* = 197)*	High risk consumption (*n* = 215)	*p*-value
Work Colleague	11.2	10.2	0.87
Wife/husband/common law partner	42.6	40.5	0.69
Relative	38.1	54.9	0.001
Friend	35.5	50.7	0.002
Medical doctor	24.9	28.4	0.44
Other health professional	3.6	1.7	0.36

*Risk consumption: Males: AUDIT-C score 4-8; Females: score 3-7; High risk consumption: Males: AUDIT-C score 9-12; Females: score 8-12

Participants who said that a medical doctor or other health professional had expressed concern about their drinking or suggested they cut down (*n* = 117) were asked if some sort of support was provided and, if so, what type of support it was. As there were no significant differences (*p* > .05) between at risk and high risk drinkers on these questions, the results are summarized for the combined drinker groups. Of those participants who said that a health professional had expressed concern, all had advised the participant to cut down and 45.3% (*n* = 53) had provided some sort of support. Of those who said that some sort of support was provided (*n* = 53), 69.8% said that brief advice was provided, 56.6% were given a pamphlet, 52.8% received some type of individual or group counselling, 34% were referred to a specialist, 15.1% were prescribed medication, and 49.1% had a follow-up appointment arranged with a health care professional.

Table 3 displays the proportion of somebody expressing concern, and of a health worker expressing concern, by risk drinking groups and whether any treatment was accessed in the last year. While there was overlap in whether participants said that someone had expressed concern about their drinking and any reports of treatment use in the past year, the concordance was not complete, particularly in the at risk group.

**Table 3:** Proportion receiving past year expressions of concern about their drinking between risk drinking groups and those who reported receiving or not receiving treatment in the last year

	Risk consumption*		High risk	
	No treatment (*n* = 2399)	Treatment (*n* = 166)	No treatment (*n* = 472)	Treatment (*n* = 109)
% (n) Somebody expressed concern	6.3 (152)	27.1 (45)	29.9 (141)	67.9 (74)
% (n) Health worker expressed concern	1.3 (32)	12.7 (21)	6.4 (30)	31.2 (34)

*Risk consumption: Males: AUDIT-C score 4-8; Females: score 3-7; High risk consumption: Males: AUDIT-C score 9-12; Females: score 8-12

## Discussion

Most participants did not receive expressions of concern about their drinking, although they were more common among high risk than at risk participants. For those who did receive expressions of concern, the most likely source was from friends and family members with only about one quarter saying that a health professional had voiced concern. Interestingly, while expressions of concern from family and friends were more common among participants in the high risk group than in the at risk group, there were no significant differences (*p* > .05) between risk groups in the proportion receiving expressions of concern from health professionals or from the participants’ marital partner. For health professionals, this lack of difference between risk groups could reflect that all at risk and high risk drinkers deserve intervention. However, it is also notable that the majority of participants did not report receiving expressions of concern about their drinking from a health professional, even among the high risk group [15, 16].

For participants who said that they had received expressions of concern from a health professional, all stated that they had received advice to cut down and about half had provided some additional support (the most common being brief advice, given a pamphlet, or individual counselling). While there were no significant differences between risk groups on the type of support provided, this could in part reflect that relatively small sample size (*n* = 53) despite the large number of at risk and high risk participants recruited.

While there was an overlap in participants who reported receiving expressions of concern about their drinking and those who reported receiving treatment in the past year, the concordance was far from perfect. This is troubling as it is hard to believe that participants could have accessed treatment and have not received at least some sort of expression of concern or suggestion to cut down from some type of health professional. Such lack of overlap may indicate that participants make a distinction between use of treatment and receipt of expressions of concern, whether due to some element of participants’ treatment experience (e.g., waiting lists) or to their own motivation for seeking treatment (e.g., autonomous versus intrinsic motivation).

## Conclusions

Even though participants may have different perception of what they will consider as expression of concern by a health care professional, components of brief intervention (advice to cut down, brief advice, counselling, etc.) were reported by participants when asked about health care professional’s concerns or advice to cut down. Therefore, while it has not been commonly used as a primary outcome [4], caution should be used when considering the AUDIT score as an outcome in a brief intervention trial, as the risk of an inflation of the scores in the group receiving the intervention is plausible. Also notable was the low prevalence with which high risk drinking participants indicated that they had received expressions of concern about their drinking from health professionals, a finding noted in other research [17]. Finally, there appears to be a wide range of sources of expressions of concerns, indicating the worth of probing for this information if a health professional is using the AUDIT as a basis for feedback in a brief intervention (e.g. collecting information on expression of concern might offer clinicians opportunities to explore ambivalence about alcohol use by exploring potential discrepancy between patient’s and its relatives’ view of its alcohol use).

The study had several limitations, primarily to do with the generalizability of the findings. The use of Mechanical Turk to recruit participants means that the sample consists of experienced survey completers. While we can think of no reason why this sample would be more likely to provide inaccurate reports than other possible samples (particularly as we took pains to reduce the chances that the participant would anticipate that they would be paid more if they provided a particular set of responses), it is possible that the experience of this sample with receiving expressions of concern about their drinking might differ from that of other at risk drinker groups. As such, the results merit replication in other samples, ideally employing methods to recruit a representative sample.

## Abbreviations

AUDIT: Alcohol Use Disorders Identification Test
AUDIT-C: Alcohol Use Disorders Identification Test Consumption subscale
RCT: Randomized controlled trial
